# A Systematic Review of Extraction Methods, Phytochemicals, and Food Applications of *Moringa oleifera* Leaves Using PRISMA Methodology

**DOI:** 10.1002/fsn3.70138

**Published:** 2025-04-28

**Authors:** Zulfa Ajrina Fitri, Farhad Ahmadi, M. Ashraful Islam, Eric N. Ponnampalam, Frank R. Dunshea, Hafiz A. R. Suleria

**Affiliations:** ^1^ School of Agriculture, Food, and Ecosystem Sciences, Faculty of Science The University of Melbourne Parkville Victoria Australia; ^2^ Agrifeed Animal Production Mill Park Victoria Australia; ^3^ Faculty of Biological Sciences The University of Leeds Leeds UK; ^4^ Centre for Sustainable Bioproducts Deakin University Waurn Ponds Victoria Australia

**Keywords:** bioactive, extraction yield, moringa leaf, phytochemicals

## Abstract

This systematic review was aimed at examining the impact of extraction methods on the phytochemical profile of *Moringa oleifera* leaves to identify the most effective extraction technique for food application. Mainly, maceration, Soxhlet extraction, and ultrasound‐assisted extraction (UAE) are reviewed in this study for their efficiency in extracting key phytochemicals in *M. oleifera* leaves. Given the rich phytochemical profile of *M. oleifera* leaves, selecting an appropriate extraction method is important in preserving their functionality and ensuring the quality of fortified or enriched food products. Adhering to PRISMA guidelines, this review found that maceration is the most efficient method for the extraction of gallic acid, which can enhance certain food textures but may also increase hardness in products such as cream cheese; Soxhlet extraction is effective in the extraction of kaempferol but slightly diminishes the sensory attributes in beverages such as malt drinks; and the UAE method is efficient in achieving the highest yield of quercetin while maintaining desirable sensory and textural properties. Overall, these findings suggest that the interaction between phytochemicals from *M. oleifera* leaves and the food matrix can affect the sensory and functional properties of the final product. Further optimization of each extraction technique is required to maximize the potential of *M. oleifera* leaf extracts in food applications.

## Graphical abstract

This PRISMA‐guided systematic review compared three extraction methods of maceration, Soxhlet, and ultrasound‐assisted extraction (UAE) to determine their impact on the phytochemical profile of *Moringa oleifera* leaves intended for food applications. The analysis suggested method‐specific differences in the yields of key bioactive compounds including gallic acid, kaempferol, and quercetin. The findings emphasize the importance of optimizing the extraction methods to increase both the sensory attributes and functional properties of food products fortified with *M. oleifera* phytochemicals.

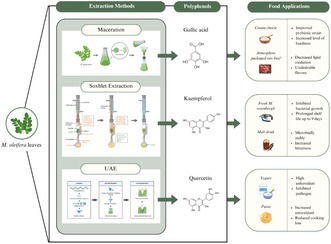

## 1 Introduction

The World Food Programme identifies food fortification as an effective strategy for improving dietary nutrition. This has led to a growing demand for research on fortified foods (Ahmad and Ahmed 2019). Food fortification involves the incorporation of minerals, vitamins, or bioactive compounds in foods in order to improve the nutritional value of the final product (Raza et al. 2024). Phytochemicals have emerged as promising fortifying agents owing to their extensive health benefits in preventing disease and addressing malnutrition (Jobby et al. 2023). Phytochemicals are bioactive compounds naturally present in plant‐based foods such as fruits, vegetables, and grains, and their inclusion in food products is associated with improved nutritional value of the final product (Abera et al. 2022).

*Moringa oleifera* is a rich source of phytochemicals and has potential for food fortification (Gopalakrishnan et al. 2016), owing to its nutritional and antioxidant properties (Hassan et al. 2021). It has been incorporated into food products, including instant porridge, ice cream, bread, and yogurt (Katmawanti et al. 2021; Ademosun 2021; Adetola et al. 2022). The phytochemical constituents abundantly present in *M. oleifera* leaves are phenolic acids, including gallic acids, and flavonoids such as quercetin and kaempferol (Vergara‐Jimenez et al. 2017). However, their incorporation into food products can have substantial impacts on some aspects. For example, incorporating *M. oleifera* leaves into beef patties can increase the protein content and shelf life due to its antioxidant activity (Al‐Baidhani et al. 2024), while their incorporation into bread formulations resulted in a less fluffy texture and a decrease in height and volume. The phytochemicals in *M. oleifera* leaves may inhibit yeast growth during fermentation, leading to a denser dough and less fluffy texture in the bread (Sengev et al. 2021). In this regard, the extraction technique may play an important role, as variations in temperature, extraction time, and pressure during leaves preparation can influence the quantity and composition of phytochemicals extracted from *M. oleifera* leaves (Bitwell et al. 2023), some of which possess antimicrobial properties (Anzano et al. 2022).

Extraction is the initial step for any plant biomolecule studies. The extraction of bioactive compounds from *M. oleifera* leaves is typically performed through three main methods: maceration, Soxhlet extraction, and ultrasound‐assisted extraction (UAE). Some extraction methods may preserve or enhance the bioactive properties of the compounds in leaves, while others may result in degradation or loss of bioactivity, which in turn affects the sensory attributes and overall quality of the final food product (Sandeep et al. 2023a). In the maceration technique, coarse plant materials are immersed in organic solvents, typically performed at low temperatures. It is crucial for preserving heat‐sensitive phytochemicals such as polyphenols (Amirullah et al. 2023). However, maceration may result in incomplete solvent penetration, leading to a lack of sensory properties in food products (Putra et al. 2023). Another technique is Soxhlet, which is highly efficient for extraction of a wide range of bioactive compounds. The continuous cycling of the solvent through the sample ensures a thorough extraction process and repeated exposure of the solid material to fresh solvent (Quitério et al. 2022). But the high temperature used during the extraction process may degrade some heat‐sensitive compounds, impacting mouthfeel (Sandeep et al. 2023a). The UAE method usually requires less time to achieve the desired bioactive compound compared to maceration or Soxhlet extraction methods. However, the optimization of parameters is required to maximize the result (Louie et al. 2020).

Each extraction method has advantages and disadvantages that can affect the yield and quality of phytochemicals extracted (Sandeep et al. 2023a). It is thus imperative to identify the most effective technique for food applications to maintain their sensory attributes and overall acceptability. However, there is still a gap in the literature on this topic, and this systematic review was designed to fill this knowledge gap by focusing on the phytochemicals present in *M. oleifera* leaves obtained through three different extraction methods and correlates these findings with current knowledge on the application of *M. oleifera* leaves in food products. By comparing the effects of all described extraction methods, for example, maceration, Soxhlet extraction, and UAE, on phytochemicals such as gallic acid and flavonoids, specifically quercetin and kaempferol, this review aims to identify the most effective extraction method for enhancing the application of *M. oleifera* leaves in food products.

## 2 Methodology

### 2.1 Data Sources and Search Strategy

**FIGURE 1:**
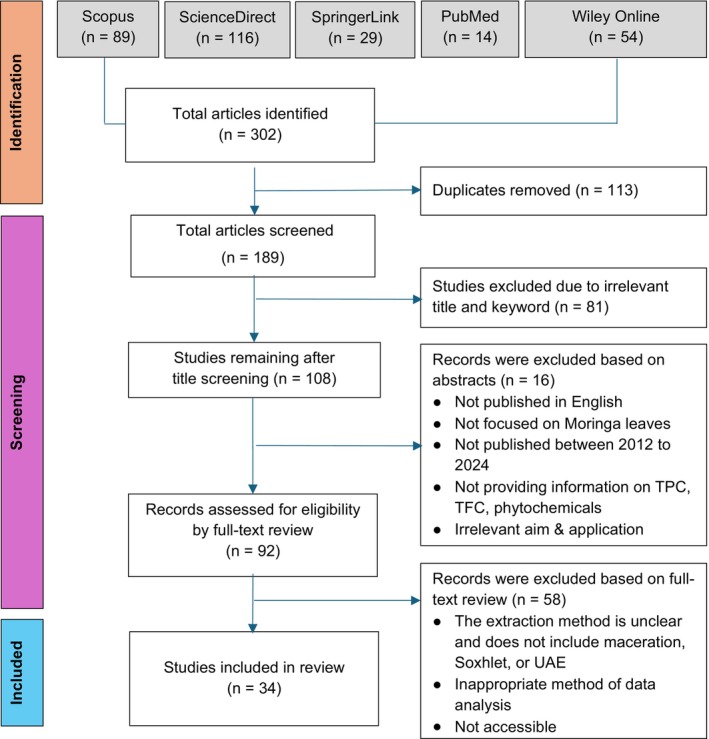
Overview of Preferred Reporting Items for Systematic Reviews and Meta‐Analyses (PRISMA) flow chart.

This systematic review was performed in accordance with the Preferred Reporting Items for Systematic Reviews and Meta‐Analyses (PRISMA) guidelines (Figure 1). The studies were collected from the following databases: ScienceDirect, Scopus, PubMed, Wiley Online, and SpringerLink. The search was conducted using the keywords “ *Moringa oleifera* leaves”, “yield”, “extraction,” “phytochemicals”, and “food application”. Duplicate entries were eliminated, and the original data was collected, compiled, and cited properly.

### 2.2 Study Selection, Inclusion, and Exclusion Criteria

The selection criteria used to assess full articles relied on the following required details: Initially, articles published in English from 2012 to 2024 were considered. Only research articles focused on the leaves part of *M. oleifera* were included, while articles examining any other parts were excluded. The articles needed to explain the specific extraction methods of *M. oleifera* leaves, including maceration, Soxhlet extraction, and UAE methods. Further, the articles should include information about total polyphenol content (TPC), total flavonoid content (TFC), and some key phytoconstituents, including gallic acid, quercetin, and kaempferol. Evidence regarding the application of *M. oleifera* in food products was also required. Articles addressing agricultural material, or veterinary applications, as well as other review articles, meta‐analyses, books, or articles unable to meet the inclusion criteria were excluded. All collected articles were screened based on the title, abstract, and subsequent full‐text analysis, adhering to the PRISMA flow diagram (Figure 1).

### 2.3 Data Extraction

To assess the three extraction methods for *M. oleifera* leaves and their relevance to food applications, two structured data collection tables were developed. The first table (Table 1) outlined the key parameters of the extraction method, including the solvent used, temperature, time, and how these parameters influenced the yield, TPC, TFC, and phytoconstituents in *M. oleifera* leaf extracts. The second table (Table 2) summarizes the existing knowledge about the incorporation of *M. oleifera* leaves in food products, highlighting both the benefits and potential limitations associated with their use in food formulations.

**TABLE 1:** Bias analysis of *in vitro* studies on selected articles on *M. oleifera* leaves.

Reference	Score						Total score	Risk
	Item 1	Item 2	Item 3	Item 4	Item 5	Item 6		
Abera et al. (2022)	1	1	1	1	2	1	7	M
Al‐Ghanayem et al. (2022)	2	1	2	2	2	2	11	L
Bennour et al. (2021)	2	1	2	2	2	2	11	L
da Silva et al. (2022)	2	2	2	1	2	1	10	L
García‐Beltrán et al. (2020)	2	1	1	1	2	2	9	M
Gomes, Leitão, et al. (2023)	2	2	2	2	2	2	12	L
Gomes, Albuquerque, et al. (2023)	2	2	2	2	2	2	12	L
Karim et al. (2018)	2	2	2	2	1	2	11	L
Kashaninejad et al. (2021)	2	2	2	2	2	2	12	L
Khalid et al. (2023a)	2	2	2	2	1	2	11	L
Khalid et al. (2023b)	2	2	2	2	2	0	10	L
Mabrok and Mohamed (2019)	2	1	2	2	2	2	11	L
Mabrouki et al. (2020)	2	2	2	2	2	2	12	L
Mahdi et al. (2016)	2	2	2	2	2	2	12	L
Mehganathan et al. (2024)	2	2	2	2	2	2	2	L
Mohamed et al. (2018)	2	2	2	1	1	1	9	M
Pakade et al. (2013)	2	2	2	2	2	2	12	L
Pereira et al. (2021)	2	2	2	2	2	2	12	L
Pollini et al. (2020)	2	2	2	1	1	1	9	M
Rastogi et al. (2024)	2	2	2	2	2	2	12	L
Sandeep et al. (2023a)	2	2	2	2	2	2	12	L
Sandeep et al. (2023b)	2	1	2	1	2	2	10	L
Sandeep et al. (2022)	2	1	1	1	2	2	9	M
Setyani et al. (2023)	2	2	2	2	2	2	12	L
Shah et al. (2015)	2	1	1	2	2	1	9	M
Shervington et al. (2018)	2	2	2	2	2	2	12	L
Sulastri et al. (2018)	2	2	2	2	2	2	12	L
Thangaiah et al. (2024)	2	2	2	2	2	2	12	L
Thomas et al. (2020)	2	1	1	2	2	1	9	M
Virk et al. (2023)	2	1	2	2	2	2	11	L
Vongsak et al. (2013)	2	1	2	2	2	2	11	L
Wu et al. (2020)	2	2	2	2	2	2	12	L
Zhao and Zhang (2013)	2	2	2	2	2	2	12	L
Zhu et al. (2020)	2	2	2	2	2	2	12	L

*Note:* Item 1 = clearly stated aim. Item 2 = accurate experimental design. Item 3 = identification and evaluation of sample. Item 4 = comparability and reproducibility. Item 5 = other bias. Item 6 = adequate statistical analysis. Item score 0 = not reported/described. Item score 1 = unclear/inadequately assessed. Item score 2 = briefly described/adequately assessed.

Abbreviations: L, low risk; M, moderate risk.

**TABLE 2:** Effects of extraction methods on yield and phytochemicals in *M. oleifera* leaves.

Items	Extraction methods	Solvent	Time	Temperature	Content	References
Yield	Maceration	70% ethanol	20 h	40°C	12.65%	Sandeep et al. (2023b)
	Maceration	70% ethanol	24 h	RT	15.55%	Sandeep et al. (2023a)
	Maceration	Water	72 h	40°C	13.23%	Sandeep et al. (2023b)
	Soxhlet	70% ethanol	16 h	40°C	6.60%	Al‐Ghanayem et al. (2022)
	Soxhlet	Methanol	16 h	NA	18.56%	Zhao and Zhang (2013)
	Soxhlet	n‐Hexane	8 h	78°C	9.30%	Pereira et al. (2021)
	UAE	70% ethanol	30 min	30°C	21.79%	Sandeep et al. (2023a)
	UAE	14.6% ethanol	5 min	30°C	31.0%	Thangaiah et al. (2024)
	UAE	70% ethanol	30 min	35°C	23.41%	Sandeep et al. (2022)
TPC	Maceration	70% ethanol	24 h	RT	130.16 mg QE/g	Bennour et al. (2021)
	Maceration	Water	24 h	NA	101.81 mg GAE/g	Vongsak et al. (2013)
	Maceration	70% ethanol	72 h	RT	132.30 mg CAE/g	Virk et al. (2023)
	Soxhlet	70% ethanol	16 h	40°C	123.17 mg QE/g	Bennour et al. (2021)
	Soxhlet	70% ethanol	20 h	NA	45.5 mg CAE/g	Virk et al. (2023)
	Soxhlet	50% ethanol	20 h	NA	44.6 mg CAE/g	Virk et al. (2023)
	UAE	70% ethanol	30 min	30°C	144.52 mg QE/g	Sandeep et al. (2023a)
	UAE	Methanol	5 min	NA	149.70 mg GAE/mL	García‐Beltrán et al. (2020)
	UAE	70% ethanol	30 min	35°C	144.90 mg GAE/mL	Sandeep et al. (2022)
TFC	Maceration	70% ethanol	24 h	RT	18.62 mg GAE/mL	Bennour et al. (2021)
	Maceration	80% ethanol	20 h	RT	26.33 mg QE/g	Mahdi et al. (2016)
	Maceration	50% methanol	48 h	45°C	12.06 mg QE/g	Wu et al. (2020)
	Soxhlet	70% ethanol	16 h	40°C	17.90 mg GAE/mL	Bennour et al. (2021)
	Soxhlet	70% ethanol	20 h	NA	24.5 mg IQE/g	Virk et al. (2023)
	Soxhlet	50% ethanol	20 h	NA	12.7 mg IQE/g	Virk et al. (2023)
	UAE	70% ethanol	30 min	35°C	24.23 mg GAE/mL	Sandeep et al. (2023a)
	UAE	Deep eutectic solvent	30 min	40°C	48.90 mg RE/g	Setyani et al. (2023)
	UAE	50% ethanol	45 min	35°C	56.63 mg QE/g	Thomas et al. (2020)
Gallic acid	Maceration	70% ethanol	72 h	RT	150.0 μg/g	Mabrok and Mohamed (2019)
	Maceration	70% methanol	72 h	NA	51.20 μg/g	Vongsak et al. (2013)
	Soxhlet	Water	20 min	NA	46.00 μg/g	da Silva et al. (2022)
	Soxhlet	99% ethanol	6 h	60°C	23.00 μg/g	Zhu et al. (2020)
	UAE	100% methanol	40 min	20°C	4.24 μg/g	Rastogi et al. (2024)
	UAE	50% methanol	30 min	25°C	11.20 μg/g	Sulastri et al. (2018)
Quercetin	Maceration	96% ethanol	72 h	RT	64.80 μg/g	Khalid et al. (2023b)
	Maceration	70% ethanol	72 h	RT	45.01 μg/g	Pakade et al. (2013)
	Soxhlet	Acidified methanol	14 h	90°C	21.50 μg/g	Mehganathan et al. (2024)
	Soxhlet	99% ethanol	6 h	60°C	13.00 μg/g	Zhu et al. (2020)
	UAE	60% ethanol	14 min	48°C	55.56 μg/g	Pollini et al. (2020)
	UAE	50% methanol	35 min	45°C	65.40 μg/g	Mabrouki et al. (2020)
Kaempferol	Maceration	Water	24 h	RT	1.84 μg/g	Vongsak et al. (2013)
	Maceration	Butanol	24 h	RT	1.02 μg/g	Shervington et al. (2018)
	Soxhlet	Acidified methanol	14 h	90°C	79.40 μg/g	Mehganathan et al. (2024)
	Soxhlet	HCl 0.1 M	3 h	90°C	84.93 μg/g	Kashaninejad et al. (2021)
	UAE	50% methanol	35 min	45°C	30.10 μg/g	Mabrouki et al. (2020)
	UAE	20% ethanol	NA	50°C	16.90 μg/g	Pareek et al. (2023)

Abbreviations: CAE, chlorogenic acid equivalent; GAE, gallic acid equivalent; IQE, isoquercetin equivalent; NA, information not given; QE, quercetin equivalent; RE, rutin equivalent; RT, room temperature.

### 2.4 Risk of Bias Assessment

A bias analysis for in vitro studies was conducted in accordance with the Methods Guide for Comparative Effectiveness Reviews as described by Viswanathan et al. (Viswanathan et al. 2008). The assessment included 6 items (Table 1): clearly stated aim considered as item 1, accurate experimental design as item 2, sample identification and evaluation as item 3, comparability and reproducibility as item 4, other bias as item 5, and adequate statistical analysis as item 6. Item 1 was scored 0 if the article's aim did not correspond to the research, 1 if the aim was unclear, and 2 if the aim was clearly reported. Item 2 (experimental design) was scored 0 if the experimental design was not described, 1 if there was unclear experimental design, and 2 if the experimental design was reported in detail and accurately. Item 3 (sample identification and evaluation) was scored 0 if any polyphenols were not reported, 1 if the value of some properties like TPC and TFC were reported, and 2 if the phytochemical constituents, including gallic acid, kaempferol, and quercetin, were reported in detail. Item 4 (comparability and reproducibility) was scored 0 if the extraction methods required non‐lab standards, 1 if the methods were less complex but had low reproducibility, and 2 if the methods could be easily practiced in standard labs with consistent results. Item 5 (other bias) was scored 0 if the abstract, methods, and conclusions were poorly or not described, 1 if they were too brief, and 2 if they were adequately detailed. Item 6 (statistical analysis) was scored 0 if not performed, 1 if partially done, and 2 if detailed (e.g., standard deviation). The total score determined the risk category: 0–6 for high risk, 7–9 for moderate risk, and 10–12 for low risk.

## 3 Results and Discussion

### 3.1 PRISMA Flow Selection

**FIGURE 2:**
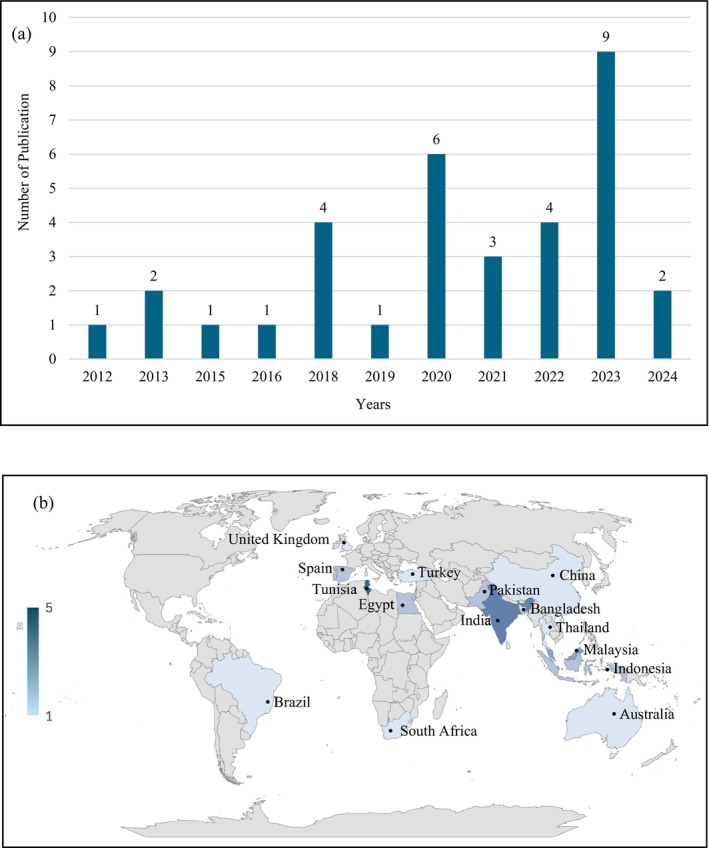
Distribution of selected studies based on publication year (a) and country of origin (b).

As presented in the PRISMA flow chart (Figure 1), the initial search resulted in the identification of 301 related records through the database searches. After removing duplicates, 188 records remained. Of these, 92 articles were selected for full‐text assessment. Ninety‐six records were excluded for reasons including: they were not published in English, did not focus on Moringa leaves, did not provide information on TPC, TFC, phytoconstituents, food application, or were not published between 2012 and 2024. Following the application of pre‐determined criteria, 58 articles were removed, leaving 34 eligible articles that investigated the effect of extraction method on TPC, TFC, phytoconstituents, and food application of *M. oleifera*. The distribution of studies selected for this review shows distinctive trends across several dimensions (Figure 2). Most of the sources were published recently, especially in 2023, an indication of recent interest in the subject matter. Tunisia emerges as a major source of studies, with a substantial number of publications exploring the phytochemical composition and food application of Moringa leaves.

### 3.2 Bias Analysis

The risk of bias of in vitro studies is tabulated in Table 1. The overall bias risk analysis of the studies indicates a moderate to low risk of bias across multiple parameters.

Almost all studies are relevant and closely related to the aim of the present topic. Over 80% of studies provided detailed information on the phytochemical constituents in *M. oleifera* leaves, including gallic acid, kaempferol, and quercetin, with adequate descriptions of abstract, methods, and conclusion. More than 70% use extraction methods that are reproducible in standard laboratory settings without requiring specialized equipment, and they report robust statistical analyses. Lastly, above 65% of studies provided a detailed description of the experimental methods.

### 3.3 Effects of Extraction Methods on Yield, TPC, and TFC

**FIGURE 3:**
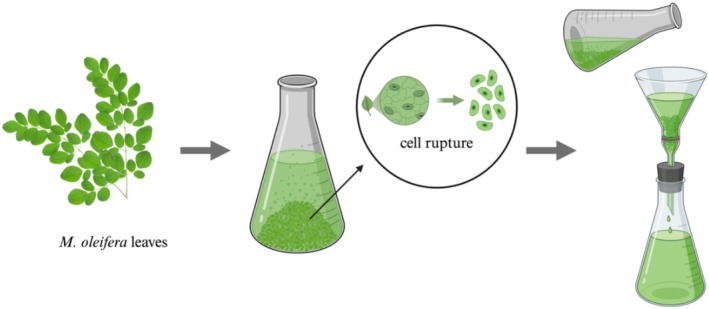
Technique of *Moringa oleifera* leaves extraction using the maceration method. The figure was created using BioRender (https://BioRender.com, accessed on 30 April 2024).

In general, *M. oleifera* crude extracts are obtained using maceration, Soxhlet, and UAE techniques. Maceration is one of the simplest methods involving immersing coarse plant materials in organic solvents such as methanol, acetone, ethanol, ethyl acetate, hexane, or water, typically at room temperature. During maceration, the plant cells are ruptured (Figure 3), allowing the bioactive components to come into contact with the solvent and be extracted (Farooq et al. 2022).

**FIGURE 4:**
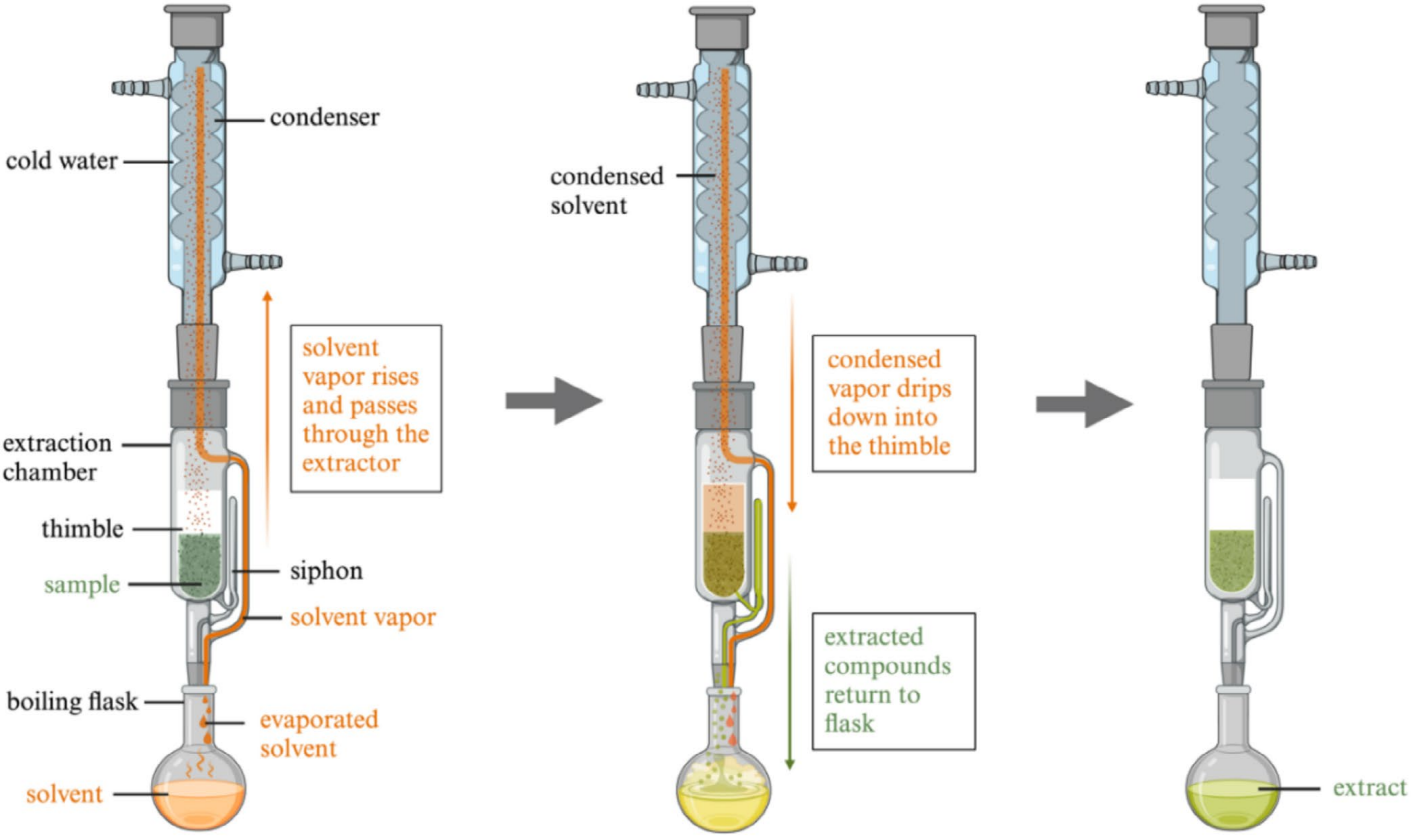
Technique of *Moringa oleifera* leaves extraction using Soxhlet extraction. The figure was created using BioRender (https://BioRender.com, accessed on 30 April 2024).

Soxhlet extraction is another widely used technique for the extraction of plant phytochemicals and is performed using a specialized apparatus known as the Soxhlet apparatus (Figure 4). In this extraction method, the solvent is heated in a round‐bottom flask, and the vapor rises to the condenser and passes through the extraction chamber. The condensed droplets then drip down into the porous thimble containing the sample. The solvent dissolves the target compounds from the plant material, gradually filling the extraction chamber. Once the level of solvent reaches above the siphon bend, the solvent returns to the round‐bottom flask, where it accumulates (Malik and Mandal 2022; Tian et al. 2016; Qin et al. 2023). The continuous cycling of the solvent through the sample ensures a thorough extraction process and repeated exposure of the solid material to fresh solvent repeatedly (Quitério et al. 2022). Compared to the maceration method, Soxhlet extraction uses less solvent and enhances solute diffusion, which accelerates the extraction process (Amirullah et al. 2023).

**FIGURE 5:**
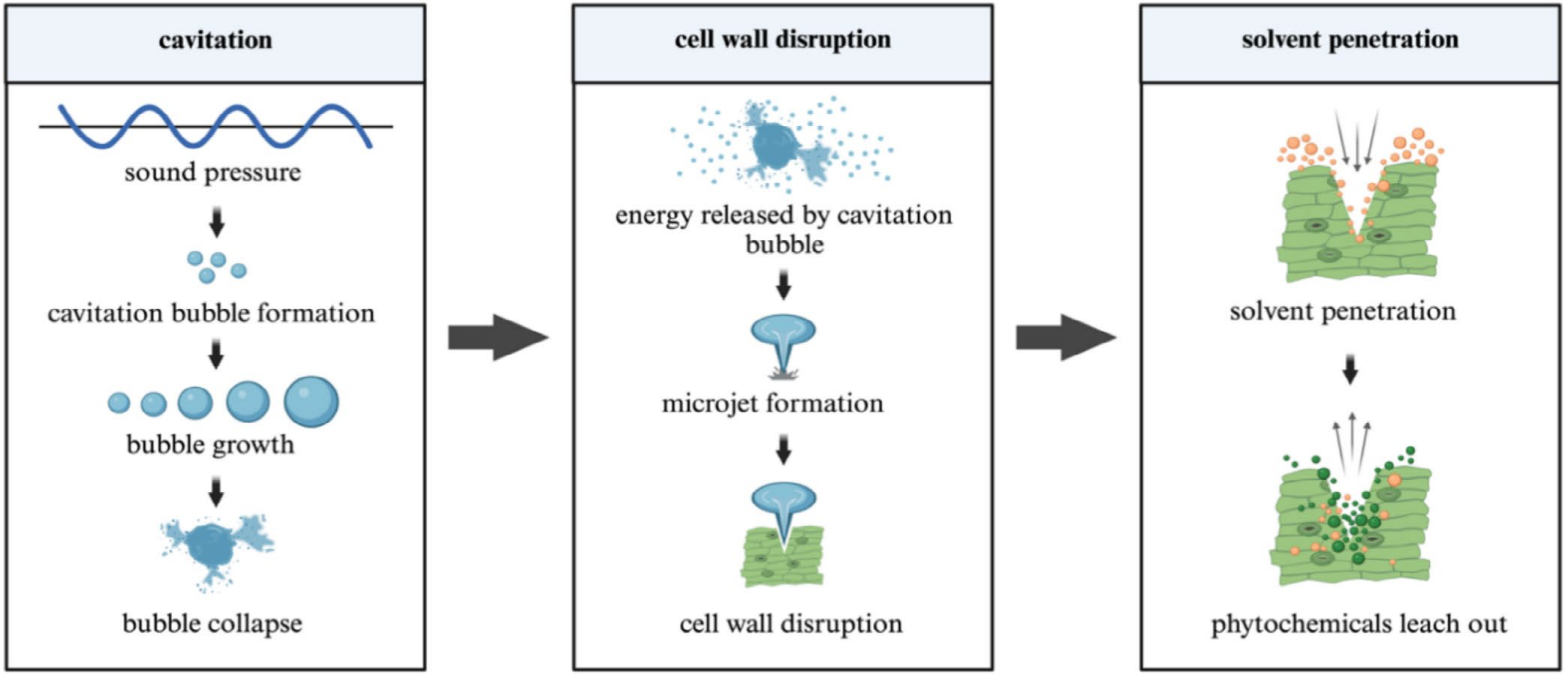
Technique of *Moringa oleifera* leaves extraction using ultrasound‐assisted extraction (UAE). The figure was created using BioRender (https://BioRender.com, accessed on 30 April 2024).

A modern and widely used technique for the phytochemical extraction in *M. oleifera* leaves is UAE. In the UAE method, high‐frequency ultrasound pulses generate localized hotspots on a macroscopic scale (Figure 5), inducing high shear stress and temperature through the generation of cavitation bubbles. These bubbles expand and then collapse, releasing energy that produces localized high temperatures and pressures. The energy released by the collapsing bubbles creates shock waves and microjets, which break down the plant cell walls. When these bubbles collapse, they generate localized high pressure and temperature, which can disrupt cell walls and enhance mass transfer. This mechanism improves solvent penetration into plant material, allowing for a more thorough extraction of polyphenols (Vernès et al. 2020). The UAE method can be more energy‐efficient than other extraction methods, as it operates at lower temperatures and achieves faster extraction rates. Owing to the enhanced extraction efficiency, UAE typically requires less time to achieve the desired yield of bioactive compounds compared to other methods such as maceration or Soxhlet extraction. Also, its capability to operate under room temperature plays a crucial role in preventing oxidation and decomposition of target natural products (Louie et al. 2020). These mechanisms make UAE the most effective method, yielding the highest TPC, TFC, and overall extract yield from *M. oleifera* leaves. Maceration ranked second in efficiency, while Soxhlet extraction produced the lowest outcomes for these parameters. The comparative results are reported in Table 2.

*M. oleifera* leaves contain various phytochemicals that are sensitive to high temperatures (Pareek et al. 2023), including phenolic acids and flavonoids. For example, gallic acid begins to degrade at temperatures above 60°C (Antony and Farid 2022), while flavonoids such as quercetin and kaempferol may experience thermal degradation at approximately 70°C (158°F) (Speisky et al. 2022). The UAE method typically operates at controlled lower temperatures (often below 50°C) (Kumar et al. 2021). This mild condition helps maintain the stability of sensitive compounds such as gallic acid, quercetin, and kaempferol. The high energy of ultrasound waves causes a faster extraction process. In this mechanism, there is a quick release of polyphenols from the plant material. The faster process may minimize the risk of degradation of sensitive compounds, thereby retaining a higher concentration of TPC.

Prior research has reported the efficacy of ultrasonication in advancing food processing and enhancing the physicochemical attributes and quality of foods. The high‐intensity ultrasound technology can potentially serve as a direct, efficient, swift, and eco‐friendly approach for the extraction of polyphenols. For example, employing a high ultrasonic intensity of 700 W was effective in the extraction of mangiferin from *Phaleria macrocarpa* fruits (Lim et al. 2019). Similarly, ultrasonication increased the TPC in sorghum flour. In these studies, a high‐energy ultrasonic device operating at 1000 W and an ultrasonic processor at 500 W were used, with the increase in free phenolic acid content attributed to the increased release of bound polyphenols (Lohani and Muthukumarappan 2021). Similarly, the combination of acid with ultrasonic extraction for *Rubus idaeus* L. enhanced the efficiency of extracting bound phenolic acids, even when using a relatively low ultrasonic power of 320 W (Wang et al. 2019).

In contrast, Soxhlet extraction requires continuous heating of the solvent, often at temperatures above 80°C (Tzanova et al. 2020). This high‐temperature exposure can rapidly degrade sensitive compounds such as quercetin and gallic acid. This leads to a lower yield of TPC and TFC. Though extraction using the maceration method preserves more sensitive compounds due to the lower temperature employed, the prolonged extraction time associated with this method may result in the gradual degradation or transformation of compounds over time, potentially reducing the yield (Shen et al. 2023).

As reported in Table 2, the extraction solvent significantly influences the extraction yield. Maceration and UAE methods resulted in higher yields as ethanol concentration increased. The polarity of ethanol allows it to effectively dissolve a wide range of compounds from the plant material (Lim et al. 2019). Polar solvents can dissolve polar compounds such as phenolic acids and flavonoids, resulting in increased efficiency of extraction (Lohani and Muthukumarappan 2021). However, in the Soxhlet extraction method, methanol produced a higher yield, likely because methanol is less prone to oxidation and degradation compared to ethanol (Wang et al. 2019). In the Soxhlet extraction method, where the solvent is continuously heated over a prolonged period, the stability of the solvent becomes crucial. Greater chemical stability of methanol makes it more suitable for extended extraction processes, resulting in minimal degradation of the solvent and higher yields of bioactive compounds.

The highest TPC value was observed with UAE when methanol was utilized as a solvent. However, direct comparison with other methods is difficult owing to the specific extraction parameters employed in the study. Stability of methanol facilitates superior extraction of polyphenols at high temperatures, resulting in a higher TPC (Wang et al. 2019). It is important to consider that the exceptionally short extraction period used in this study may have influenced the extraction efficiency. This highlights the significance of extraction conditions in interpreting TPC values.

*M. oleifera* leaves macerated with ethanol exhibited higher flavonoid content compared to methanol. It is likely because ethanol is capable of dissolving both polar and non‐polar compounds, thus effective for flavonoids extraction (Tzanova et al. 2020). Moreover, ethanol has the ability to solubilize quercetin and kaempferol (Shen et al. 2023). Consequently, ethanol yields higher TFC from *M. oleifera* leaves. However, the UAE method achieved the highest TFC using lower ethanol concentrations. This may be influenced by the cavitation phenomenon induced by ultrasound, which creates intense localized pressure and temperature gradients within the solvent. These fluctuations may enhance the interactions between the solvent molecules and the solutes in the plant material (Vinatoru 2001). In the case of lower ethanol concentrations, where solvent molecules may be less prone to clustering, the cavitation‐induced turbulence and microstreaming promote more uniform and intimate contact between the solvent and the plant surface (Vinatoru 2001). This facilitates the dissolution of target compounds, such as flavonoids.

Optimal yields of polyphenols in UAE method depend on the selection of an appropriate solvent. Methanol is the preferred solvent for extracting polyphenols from *Centaurea* sp. leaves, with ethanol following closely. Conversely, ethanol is the most effective solvent for the extraction of phenolic acids from mango peels (Martínez‐Ramos et al. 2020). Similarly, the ethanolic extracts of *Laurus nobilis* exhibited the highest TPC compared to water and methanolic extracts (Rincón et al. 2019). However, it is important to note that geographical origin and plant species or cultivars may also influence the yield of extracted polyphenols (Zainol et al. 2020).

### 3.4 Effects of Extraction Methods on Phytoconstituents

**FIGURE 6:**
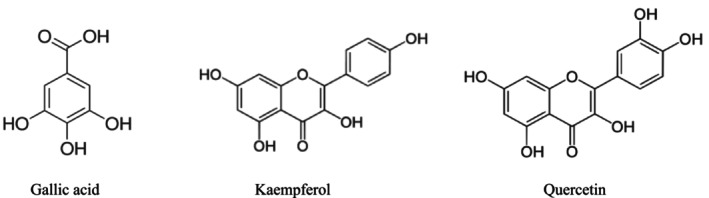
Chemical structure of phytoconstituents in *M. oleifera* leaves.

The three most abundant phytoconstituents in *M. oleifera* leaves are gallic acid, quercetin, and kaempferol. Each extraction method influenced the major phytoconstituents in *M. oleifera* leaves. *M. oleifera* leaves extracted using the maceration technique yield the highest concentration of gallic acid (Table 2). Maceration involves soaking the plant material in a solvent at room temperature or slightly higher (Subramanian and Anandharamakrishnan 2023). The solvents, typically ethanol, methanol, or water, diffuse into the plant cells, breaking down the cell wall and releasing gallic acid into the solvent (Bitwell et al. 2023). The chemical structure of gallic acid further supports this mechanism. Gallic acid contains a benzene ring with three hydroxyl groups (–OH) (Figure 6) (Charlton et al. 2023), which can form hydrogen bonds with polar solvents such as water and alcohol (Spange et al. 2022). These interactions help dissolve gallic acid into the solvent by reducing the intermolecular forces between gallic acid molecules and allowing it to disperse more easily during maceration (Park and Lee 2024). The aromatic ring in gallic acid contributes significantly to its antioxidant properties; however, its hydroxyl groups make it highly susceptible to oxidation. Exposure to heat, light, or strong mechanical stress can trigger oxidative degradation, leading to the formation of reactive oxygen species (ROS). This process not only compromises the structural integrity of gallic acid but also reduces its antioxidant efficacy, ultimately impacting its stability and functional benefits in food and pharmaceutical applications (van Lith and Ameer 2016; Qu et al. 2024). In oxidative conditions, gallic acid may undergo auto‐oxidation, leading to the formation of quinones (Tan et al. 2023) or participate in oxidative coupling reactions with other polyphenols (Kieserling et al. 2024), potentially modifying the texture, digestibility, and bioavailability of both nutrient and gallic acid (Tan et al. 2023; Sun et al. 2023). Thus, the conditions of maceration help preserve gallic acid from harsh exposure, that commonly occurs in Soxhlet and UAE methods (Bitwell et al. 2023; Gil‐Martín et al. 2022). Soxhlet extraction involves continuous boiling and condensation (Nafiu et al. 2019), while UAE uses ultrasound waves (Weggler et al. 2020). Both methods carry a higher risk of thermal or mechanical degradation of gallic acid.

*M. oleifera* leaves extracted using the Soxhlet extraction technique yield the highest concentration of kaempferol (Table 2). Kaempferol's structure contains four hydroxyl groups (Dabeek and Marra 2019) contributing to its strong antioxidant activity, anti‐inflammatory, anticancer, and neuroprotective effects (Kaur et al. 2024). This compound is poorly soluble in water but slightly polar, allowing it to dissolve well in polar solvents like ethanol (Cid‐Ortega and Monroy‐Rivera 2018), which is commonly used in Soxhlet extraction. Moreover, although high temperatures and prolonged extraction times in Soxhlet extraction may potentially degrade some compounds, kaempferol has a relatively stable molecular structure, allowing it to withstand higher temperatures without significant degradation (de Oliveira et al. 2017). Kaempferol has a typical flavonol backbone, consisting of two benzene rings connected by a heterocyclic oxygen‐containing ring (Figure 6). The presence of a C–ring and hydroxyl groups at the 3 and 4 positions allows electron delocalization, stabilizing ROS more effectively than gallic acid with a simpler structure (Jomova et al. 2023). This stability allows kaempferol to be effectively extracted using the Soxhlet method.

*M. oleifera* leaves extracted using the UAE technique yield the highest concentration of quercetin. Quercetin is a flavonol with a diphenylpropane skeleton and five hydroxyl groups (Figure 6) (Michala and Pritsa 2022). Quercetin has a catechol (ortho‐dihydroxy) structure and a C–ring heterocycle, which stabilizes radicals via electron delocalization (Carrillo‐Martinez et al. 2024). This structural feature makes quercetin one of the most potent antioxidants, surpassing kaempferol and gallic acid in antioxidative efficacy (Madiha et al. 2021). However, its high reactivity also increases its susceptibility to oxidation, making it prone to degradation under excessive heat or prolonged exposure to oxidative environments (Cao et al. 2022). The UAE method offers an advantage by operating at lower temperatures (Kumar et al. 2021) compared to Soxhlet extraction, which helps maintain the stability of quercetin, as prolonged heat (≥ 60°C) can lead to dihydroxylation or oxidation of quercetin into quinone derivatives (Bhatia et al. 2022). The presence of cavitation in UAE does not generate excess oxygen radicals that could modify the structure of quercetin. Moreover, UAE is a faster extraction method (Shen et al. 2023), reducing the exposure of quercetin to potentially harmful conditions such as extended heat or mechanical stress.

### 3.5 Effects of Extraction Methods on Food Application of *M. oleifera* Leaves

As moringa‐fortified food products continue to grow in popularity, careful consideration must be given to both their health benefits and sensory properties. The extraction method used for *M. oleifera* leaves impacts their incorporation into food products, particularly affecting sensory attributes (Table 3). Differences in extraction conditions for example, solvent type, temperature, and mechanical forces influence the yield and composition of bioactive compounds, which in turn affect the color, flavor, texture, and functional properties of the final product (Trigo et al. 2023). For example, the maceration method employs soaking plant material in a solvent (such as ethanol or water) at room temperature for an extended duration (Subramanian and Anandharamakrishnan 2023). Due to its broad extraction range, this method potentially results in a high concentration of anti‐nutrient phytochemicals, including phytate (Guan et al. 2021). These compounds, while beneficial in some contexts, can increase hardness and decrease cohesiveness in products like cream cheese, and also promote undesirable flavor in atmosphere‐packaged raw beef (Table 3).

**TABLE 3:** Benefits and drawbacks of different extraction methods for the application of *M. oleifera* leaves in food products.

Extraction Methods	Food Product	Benefits	Drawbacks	References
Maceration	Cream cheese	Improved probiotic strain Enhanced flavor Increased antimicrobial properties	An increase in leaves extract resulted in a higher level of hardness, while cohesiveness decreases	Karim et al. (2018)
	Atmosphere packaged raw beef	Reduced thiobarbituric acid Decreased lipid oxidation	Higher concentrations resulted in undesirable flavors	Gomes, Leitão, et al. (2023)
Soxhlet	Giant Freshwater Prawn ( *M. rosenbergii* )	Inhibited bacterial growth Reduced chemical quality changes Prolonged the shelf life up to 9 days Delayed melanosis	None	Gomes, Albuquerque, et al. (2023)
	Malt drink	The prepared beverages were microbially stable Prolonged shelf life up to 6 months	A slight decrease in the sensory attributes score (including color, bitterness, sweetness, flavor, and overall impression) was observed with increasing extract concentration.	Abera et al. (2022)
UAE	Yogurt	Showed higher antioxidant activities Against *Escherichia coli* and *Staphylococcus aureus* Exhibited antioxidant stability	None	Wu et al. (2024)
	Pasta	Increased antioxidant capacity Reduced the cooking loss. Extended shelf‐life	None	Bell et al. (2018)

Phytate chelates and affects the bioavailability of calcium ions in cream cheese incorporated with *M. oleifera* leaves extract (Guan et al. 2021). This ion binding results in protein–protein interaction (Guan et al. 2021), which in turn tightens the structure into a more complex matrix, increasing the hardness of the cream cheese (Guan et al. 2021) In addition, phytates present in *M. oleifera* leaves have metal‐chelating properties, binding prooxidant metal ions such as iron and copper. This reduces their availability, which could otherwise catalyze oxidative degradation, thereby lowering the oxidation level in atmosphere‐packaged raw beef modified with *M. oleifera* extract (Scarano et al. 2023). However, this strong metal‐binding property can also contribute to the development of metallic or astringent flavors, which are generally considered undesirable in food applications (Ömür‐Özbek et al. 2012).

Phenolic acids in *M. oleifera* leaves may also reduce essential amino acids, thus weakening the stabilization of protein structures in food (Karabulut et al. 2024). For example, lysine is involved in cross‐linking collagen fibers, while cysteine forms disulfide bonds stabilizing protein structure. Also, tryptophan contributes to protein stability and folding (Rawel et al. 2002). The lack of these essential amino acids results in a less cohesive and more crumbly texture in food products.

Soxhlet extraction uses organic solvents at elevated temperatures to continuously extract compounds over several cycles (Tzanova et al. 2020). However, the high temperatures and prolonged exposure associated with this method can enhance the extraction of volatile compounds, including isothiocyanates present in *M. oleifera* leaves (Wu et al. 2024; Bell et al. 2018). Isothiocyanates are known to activate bitter taste receptors (TAS2Rs), particularly TAS2R38 on the human tongue, contributing to the bitterness of malt drinks containing *M. oleifera* leaf extract (Tran et al. 2021). Moreover, polyphenols in *M. oleifera* leaves, such as kaempferol, have planar structures that may further contribute to bitterness by interacting with receptors involved in bitter taste perception (Nejabati and Roshangar 2022; Tarragon and Moreno 2020). The extended exposure to high temperature during Soxhlet extraction potentially promotes the release of bound tannins from the plant matrix (Das et al. 2020). Tannins can bind proteins in saliva and mucous membranes in the mouth, causing them to precipitate or aggregate. This leads to the dry and puckering sensation, which is associated with astringency (Soares et al. 2020).

According to Table 3, the UAE method did not show any effects or drawbacks on food products. The ultrasonic waves in the UAE method effectively break down plant cell walls, allowing for an efficient release of bioactive compounds. This process enhances the yield of desired phytochemicals without the need for prolonged exposure to solvents or high temperatures (Vernès et al. 2020). Furthermore, the UAE method can be optimized to selectively extract specific phytochemicals while minimizing the extraction of undesirable compounds that negatively affect sensory properties (Raghunath et al. 2023). The UAE often requires shorter extraction times compared to other methods (Shen et al. 2023) and can reduce the likelihood of extracting excessive amounts of polyphenols that can contribute to bitterness and astringency.

### 3.6 Work Limitation and Future Direction

This study highlights how UAE, maceration, and Soxhlet extraction methods impact the bioactive compounds in *M. oleifera* leaves. By understanding how these methods influence compound stability and yield, an optimal extraction approach can be identified to maximize bioactive retention while minimizing degradation. Further, this study explores the incorporation of *M. oleifera* extracts into various food products, such as dairy, meat, and beverages, providing practical information into how different extraction methods may affect sensory attributes, stability, and bioactivity. However, the impact of *M. oleifera* extracts on texture, flavor, and stability varies depending on food matrices.

This study also presents several limitations. The variability in solvent type, temperature, and extraction time can lead to complexity in standardizing optimal conditions, making it challenging to compare all phytoconstituents in *M. oleifera* leaves. Further, the influence of geographic origin and plant species or cultivar may cause variability, making it difficult to establish universally applicable conclusions regarding extraction protocols. This review is limited to the three most abundant phytoconstituents in *M. oleifera* leaves, without systematically evaluating the impact of various extraction methods on anti‐nutritional factors, which could influence the nutritional quality and functional properties of *M. oleifera* leaves in food application. Future research should expand on a broader range of these aspects to provide a more comprehensive understanding of the role of *M. oleifera* leaves in the food industry. Comparing studies with other extraction techniques, such as supercritical fluid extraction or microwave‐assisted extraction, along with other factors such as pH, sample‐to‐solvent ratio, and exploring various solvents with different polarities may offer opportunities to maximize the results of this review. In addition, interaction within food matrices may lead to the efficacy of phytochemicals and influence the sensory properties of food products. Therefore, the systematic evaluation of the impact of various extraction techniques on the interaction between phytochemicals and other food components could provide a better understanding of the optimization of *M. oleifera* incorporation in food products. Furthermore, investigating more examples of *M. oleifera* leaf applications in food products is essential to promote the wider adoption and greater impact of *M. oleifera* leaf extract in the food industry.

## 4 Conclusions

The extraction method used for *M. oleifera* leaves can have a substantial impact on the bioactive compounds and their benefits in food products. UAE outperforms traditional methods such as maceration and Soxhlet extraction with respect to extraction yield, TPC, and TFC. In particular, maceration is the most efficient for extracting gallic acid, UAE is best for quercetin, and Soxhlet extraction is particularly effective for obtaining kaempferol. Incorporating *M. oleifera* into food products enhances probiotic growth, flavor, shelf life, and antimicrobial and antioxidant properties. Importantly, maceration increases hardness in cream cheese, direct addition to raw beef causes undesirable flavors, and Soxhlet reduces sensory qualities in malt drinks. UAE, on the other hand, preserves sensory and textural attributes, making it a superior choice. Thus, selecting the appropriate extraction method is essential to maximize the benefits of *M. oleifera* leaves in food systems, ensuring both optimal sensory and functional qualities.

## Author Contributions

**Zulfa Ajrina Fitri:** methodology (equal), visualization (equal), writing – original draft (equal). **Farhad Ahmadi:** conceptualization (equal), data curation (equal), supervision (equal), visualization (equal), writing – review and editing (equal). **M. Ashraful Islam:** validation (equal), writing – review and editing (equal). **Eric N. Ponnampalam:** validation (equal), writing – review and editing (equal). **Frank R. Dunshea:** resources (equal), validation (equal), writing – review and editing (equal). **Hafiz A. R. Suleria:** conceptualization (equal), funding acquisition (equal), project administration (equal), resources (equal), supervision (equal), validation (equal), writing – review and editing (equal).

## Ethics Statement

The authors have nothing to report.

## Conflicts of Interest

The authors declare no conflicts of interest.

## Data Availability

The authors have nothing to report.
